# Spatio-Temporal Variation of Core and Satellite Arbuscular Mycorrhizal Fungus Communities in *Miscanthus giganteus*

**DOI:** 10.3389/fmicb.2016.01278

**Published:** 2016-08-22

**Authors:** Christopher J. Barnes, Caitlin A. Burns, Christopher J. van der Gast, Niall P. McNamara, Gary D. Bending

**Affiliations:** ^1^School of Life Sciences, University of WarwickCoventry, UK; ^2^NERC Centre for Ecology and HydrologyWallingford, UK; ^3^NERC Centre for Ecology and Hydrology – Lancaster Environment CentreLancaster, UK

**Keywords:** Glomeromycota, fungi, *Miscanthus*, core, satellite, arbuscular mycorrhizal fungi, spatio-temporal variation

## Abstract

Arbuscular mycorrhizal fungi (AMF) are a group of obligate plant symbionts which can promote plant nutrition. AMF communities are diverse, but the factors which control their assembly in space and time remain unclear. In this study, the contributions of geographical distance, environmental heterogeneity and time in shaping AMF communities associated with *Miscanthus giganteus* (a perennial grass originating from south-east Asia) were determined over a 13 months period. In particular, the community was partitioned into core (abundant and persistent taxa) and satellite (taxa with low abundance and persistence) constituents and the drivers of community assembly for each determined. β-diversity was exceptionally low across the 140 m line transects, and there was limited evidence of geographical scaling effects on the composition of the core, satellite or combined communities. However, AMF richness and community composition changed over time associated with fluctuation within both the core and satellite communities. The degree to which AMF community variation was explained by soil properties was consistently higher in the core community than the combined and satellite communities, suggesting that the satellite community had considerable stochasticity associated with it. We suggest that the partitioning of communities into their core and satellite constituents could be employed to enhance the variation explained within microbial community analyses.

## Introduction

Rhizosphere fungi play a major role in terrestrial ecosystems, shaping plant community structure and ecosystem function though parasitism, mutualism, and saprophytism (Berendsen et al., 2012). Arbuscular mycorrhizal fungi (AMF) are one of the most studied microbial inhabitants of the rhizosphere (Verbruggen et al., 2012). These fungi, from the phylum Glomeromycota, are obligate symbionts, which can form mutualistic symbioses with most plant species (Gosling et al., 2013). AMF assimilate resources from the soil, particularly phosphorus and other nutrients, which are traded with the plant in return for carbon-based metabolites (Kiers et al., 2011; Orwin et al., 2011). Consequently AMF can be a significant sink of plant assimilates, and can have far reaching effects within ecosystems, influencing nutrient cycles, soil stability and water retention (Kucey, 1987; Hodge et al., 2001; Crow et al., 2009).

A wide range of factors are known to affect the specific composition of AMF communities which assemble in the root zone. AMF communities can vary greatly over time, particularly over seasonal time frames (Escudero and Mendoza, 2005; Dumbrell et al., 2009; Pereira e Silva et al., 2012; Bennett et al., 2013). This seasonality in AMF communities may be driven directly through changing abiotic factors associated with annual cycles, such as temperature and rainfall, or indirectly through changes in carbon availability from host plants (Dumbrell et al., 2011). However, more long-term changes in AMF community composition over years and decades may also occur in response to plant age (Daniell et al., 2001; Husband et al., 2002) and changes in aboveground biodiversity (van der Heijden et al., 1998; Wardle et al., 2004).

Arbuscular mycorrhizal fungi (AMF) communities also show significant spatial variability, responding to factors such as soil properties (Dumbrell et al., 2009; Hazard et al., 2013), vegetation and climate (Treseder et al., 2004; Tedersoo et al., 2012; Peay et al., 2013). Although some fungi have far-reaching dispersal patterns, many may face the same barriers to dispersal as larger organisms, including mountains and oceans, anthropogenic pressures, and ecological factors (Treseder and Cross, 2006; Tedersoo et al., 2014). These spatial scaling effects have been demonstrated for fungi and AMF over local, landscape and continental geographic distances (Tedersoo et al., 2003; Green et al., 2004; Lilleskov et al., 2004; Peay et al., 2007; van der Gast et al., 2011), although some studies have found limited evidence for dispersal limitation affecting AMF community assembly over regional geographical scales (An et al., 2008; Hazard et al., 2013).

The majority of microbial biogeographical studies analyze spatial or temporal scaling alone, with few considering both together (Matsuda and Hijii, 1998; Courty et al., 2008; Pereira e Silva et al., 2012). However, since microbial communities are known to display great temporal and spatial variation (Bell, 2010; Barnes et al., 2016), these variables should be considered together, and the relative effects of each elucidated.

Communities can be divided into ‘core’ species, which are locally abundant and regionally persistent, and ‘satellite’ species which are both regionally and locally rare (Hanski, 1982; Magurran, 2007). Magurran and Henderson (2003) hypothesized that in macro-organisms, core species are well-adapted to surroundings, whereas, satellite species are under limitations of dispersal. This approach has proved to be a useful tool to understand ecological principles shaping communities of macro-organisms (Pärtel et al., 2001; Unterseher et al., 2011b; Supp et al., 2015) but has only infrequently been implemented in analyses of microbial communities (Ulrich and Zalewski, 2006; Unterseher et al., 2011a; Rogers et al., 2013; Lindh et al., 2015).

*Miscanthus giganteus* is a perennial grass that has been widely grown as a bioenergy crop within Europe (Kahle et al., 2001). Plantations remain untilled throughout their lifetime, thus soil communities can develop over several decades, in a soil matrix which remains relatively undisturbed (Christian et al., 2008). Diverse AMF communities have been found to associate with *Miscanthus* grasses (An et al., 2008). *Miscanthus* plantations provide an excellent system to study the spatial and temporal scaling of AMF, the homogenous plantations limiting confounding effects of disturbance, host species and genotypic variation on AMF communities (Corredor et al., 2014).

The aim of this study was to investigate the relative importance and interactions of spatial and temporal drivers of community assembly in the AMF communities of *M. giganteus*, with a particular focus on the assembly of core and satellite communities.

## Materials and Methods

### Study Site and Root Sampling

The study site was a commercial perennial *M. giganteus* bioenergy plantation, which was established in 2006 near Lincoln, UK. Prior to growing *M. giganteus*, the field had been historically cropped with an oilseed rape-wheat rotation. The *M. giganteus* was planted at a density of 10,000 rhizomes ha^-1^. There was no nitrogen fertilization applied during or subsequent to establishment. The soil was a sandy loam with 53% sand, 32% silt, and 15% clay.

**FIGURE 1:**
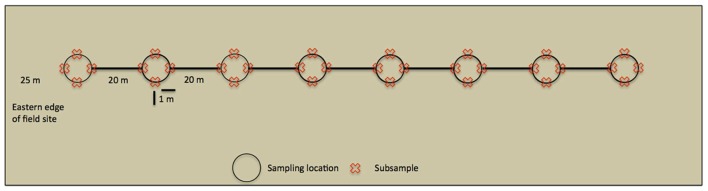
**Overview of a line transect.** Sampling started 25 m within the field site to avoid edge effects, whilst sampling locations were 20 m apart. At each sampling location 4 cm × 15 cm deep soil cores were taken (subsamples) and analyzed independently.

Within the UK, *M. giganteus* growth is most rapid in spring and early summer, although maximum height is not reached until September or October (Christian et al., 2008). Soil samples were collected on 6th October 2010, and 21st June, 9th August and 10th October 2011, in order to capture AMF community development over a complete growing season, and to allow comparison of late growth stage communities across 2 years. Sampling was performed along line transects (**Figure 1**), starting at the western edge of the field site and heading due east. An initial sampling site was located 25 m into the field to avoid edge effects, and each of the seven subsequent sampling sites was located at 20 m intervals along the transect. At each sampling site, four subsamples were taken 1 m from the central position in north, south, east, and west directions. In order to avoid resampling previously disturbed sites, entire transects were shifted 3 m east at each time point.

At each subsampling position, a soil core (0–15 cm) was collected and stored at 4°C for a maximum of 1 week prior to processing. *Miscanthus* produces a highly branched, dense network of roots growing from a rhizome. We collected roots under approximately 2 mm from the soil using tweezers. Healthy white roots were separated from darker senescent *Miscanthus* roots, and adhering soil was washed off using deionized water. The roots were cut to 1 cm lengths, thoroughly mixed, and stored at either -20°C, or in 20% (v/v) ethanol at 4°C, for molecular and staining analyses respectively.

### Soil Analysis

Soil was oven dried at 60°C prior to undergoing analysis, as previously described (Barnes et al., 2016). Briefly, soil pH was measured with a Russell model RL 150 pH meter, in a solution ratio of 1:5 with deionized water. NO_3_^-^ and NH_4_^+^ were extracted from soil with 0.5 M K_2_SO_4_ and quantified using a Foss FIAStar 5000 flow injection analyser. % C and % N were measured by combustion using a Leco CN2000 analyser. Available P was extracted with 0.5 M NaHCO_3_, while available K and Mg were extracted with 1 M NH_4_NO_3_. Available P, K, and Mg were subsequently quantified using a Jobin Yvon Ultima 2 inductively coupled plasma optical emission spectrometer.

### Mycorrhizal Staining and Colonization

Roots were stained to visualize AMF and to calculate root length colonization (RLC) by AMF (Grace and Stribley, 1991). RLC was estimated in each sample using 10 fields of view from 10 roots, using methods previously described (Brundrett et al., 1996).

### DNA Extraction and PCR

Roots were washed, blotted dry, and 200 mg added to lysis tubes provided with the PowerSoil DNA isolation kit (MP Biomedicals, Cambridge, UK), and mechanically lysed in a TissueLyser (QAIGEN, UK) using three separate 30 s pulses at 30 Hz, before undergoing extraction as per manufacturers instructions. 18S rDNA fragments were amplified using the AMF specific primers AML1 (5′-ATCAACTTTCGATGGTAGGTAGGATAGA-3′) and AML2 (5′-GAACCCAAACACTTTGGTTTCC-3′) fluorescently labeled with 6-FAM (Lee et al., 2008). AMF PCR cycle conditions were as follows: 94°C 5 min, 40 cycles of 94°C 1 min, 57°C 1 min, 72°C 1 min, then 72°C 10 min, 12°C. PCR products were purified using a QIA-quick PCR purification kit.

### Clone Library and Processing

To determine the most efficient restriction enzymes for terminal restriction fragment length polymorphism (TRFLP) analysis, and confirm specificity of the AMF PCR, AMF community 18S rRNA genes were sequenced. Two clone libraries established, one for the October-10 transect and another for June-11. For each library, 1 μl of equilibrated (25 ng/μl) DNA from each of the 32 subsamples was combined to produce a pooled sample. 18S rDNA was amplified from each pooled sample using the method described above, with unlabeled AML1 and AML2 primers. PCR products were purified using QIAquick Gel Extraction Kit (QIAGEN House, Sussex, UK), following the manufacturer’s protocol. Cloning was carried out using the QIAGEN PCR cloning plus kit (QIAGEN House, Sussex, UK), and TempliPHI Amplification kit (GE Healthcare Ltd, UK), following manufacturer’s protocols. Products were sequenced on an Applied Biosystems 3130X1 automated capillary sequencer. Sequences were quality checked, leaving 84 sequences in October-10 and 87 sequences in June-11, which were then aligned, and used for hierarchical clustering at 99% similarity using DNASTAR 9 (Lasenger, Madison, WI, USA). A total of seven operational taxonomic units (OTUs) were identified and taxonomy was assigned by BLAST searching against the nucleotide collection (nr/nt) database of the NCBI. Sequence accession numbers (KU937164, KU937170, KU937161, KU937154, KU937168, KU937155, KU937150), relative abundances and taxonomy are given in Supplementary Table S1.

Restriction enzymes were chosen by fragmenting clone library sequences *in silico* with all restriction enzymes listed on the bioperl Restriction Enzyme Mapping Application (REMA) http://bioperl.macaulay.ac.uk. The restriction enzyme which generated the highest terminal restriction fragment (TRF) richness from the clone library, *Ase*1 (New England Biolabs, UK), was chosen for AMF 18S rDNA. However these fragments could not be subsequently linked to specific TRFs, as found in other studies (Gosling et al., 2013).

### Fragment Length Analysis

Terminal restriction fragment length polymorphism was performed on the 6-FAM labeled AMF community PCR products to assess diversity of AMF. TRFLP reactions contained 300 ng of PCR product, 10% buffer and two units of restriction enzyme, made up to 30 μl with pure water. Samples were incubated for 4 h at 37°C, followed by 20 min at 65°C to denature enzymes. Mixtures of 1 μl digestion product, 9.85 μl formamide, and GeneScan^TM^ Size Standard (LIZ^®^ 1200) were denatured for 5 min at 95°C, cooled on ice, and analyzed on an Applied Biosystems 3130X1 automated capillary sequencer. TRF profiles were analyzed using GeneMarker v1.8 (SoftGenetics, Stat College, PA, USA) and characterized by peak area. TRFs above 1% relative abundance in a given sample, and between 100 and 550 bp, were included in the analysis.

### Partitioning of Core and Satellite Communities

**FIGURE 2:**
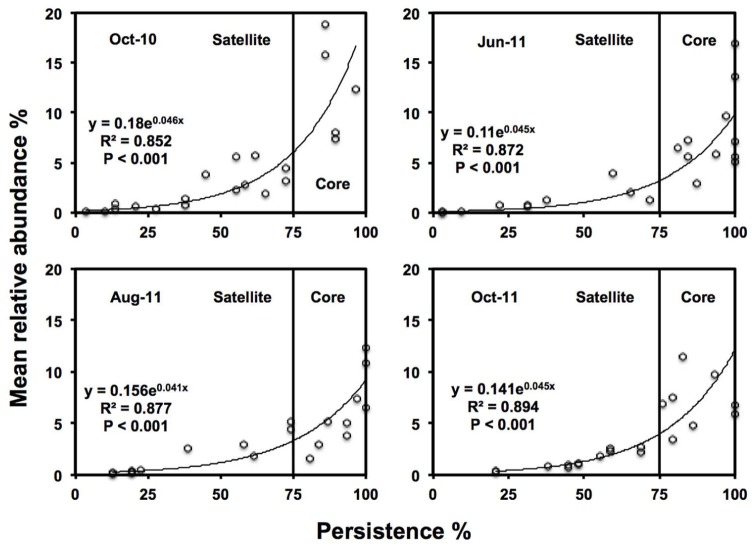
**Persistence and abundance of the twenty-one AMF TRFs, at each sampling time.** Where the vertical line is the threshold for ‘core’ taxa at 75% persistence.

‘Core’ TRFs, were differentiated from ‘satellite’ TRFs using a persistence threshold (**Figure 2**; Unterseher et al., 2011b; Siqueira et al., 2012). At every time point, persistence of each TRF (i.e., the number of samples it was found in) was plotted against relative abundance. TRFs found in >75% samples were classified as core taxa, since this approximately the point of inflection of the exponential curve in which TRF abundance showed substantially higher persistence than TRF found in <75% of samples. Those TRF found in <75% of samples, were classified as satellite (low abundance and persistence).

### Statistical Analyses

Multiple pairwise *t*-test were performed to assess differences in α-diversity (i.e., TRF richness) of the combined, core and satellite AMF communities, and also to compare RLC and soil properties at each time point. β-diversity within the combined, core and satellite communities was assessed by constructing distance-decay relationships (DDRs) for each transect using the power law equation *S* = c*D*^d^, where *S* = pair-wise similarity for any two samples using Jaccard (*S*_J_) binary (i.e., presence/absence) indices of similarity, *c* = constant, *D* = distance in meters between pair-wise samples (created via a Euclidean distance matrix), and *d* = rate of decay in similarity (Green et al., 2004). A Bray–Curtis similarity matrix was constructed for soil nutrients (*N*) before partial Mantel statistics were performed to further test for an independent geographical distance effect for each transect. In these analyses, the community matrix for each season was correlated against the distance matrix, whilst the soil compositional matrix was used to compensate for changing soil properties using the equation r(*SD.N*) for each season (Legendre and Fortin, 1989). Jaccard binary dissimilarity matrices, Euclidean distance matrices and Bray–Curtis matrices were constructed using the vegan package within *R* (Oksanen et al., 2016).

To determine whether communities were stable across time, the variability between the whole, core and satellite AMF composition (Jaccard presence/absence similarity matrixes of TRF data) at each sampling time was analyzed using an analysis of similarity (ANOSIM), and similarity percentages (SIMPER) calculated using Primer6 (version 1.0.2; Primer-E, Ivybridge, UK). Community matrices were performed using data on a presence/absence basis to limit the effects of primer bias on community analyses (Anderson et al., 2003). Further tests of spatial variation were performed via PERMANOVA using the adonis function within the vegan package of *R* (Oksanen et al., 2016), with community data from TRFLP analysis again transformed into Jaccard similarity indexes (using presence/absence data). These indices underwent analysis against soil properties (pH, C, N, NH_4_, NO_3_, P, K, Mg), using the sampling site (each consisting of four subsamples) as the strata in the individual transect analyses, and the date transects were performed in the combined analyses across all sampling times.

## Results

### Soil Properties

**FIGURE 3:**
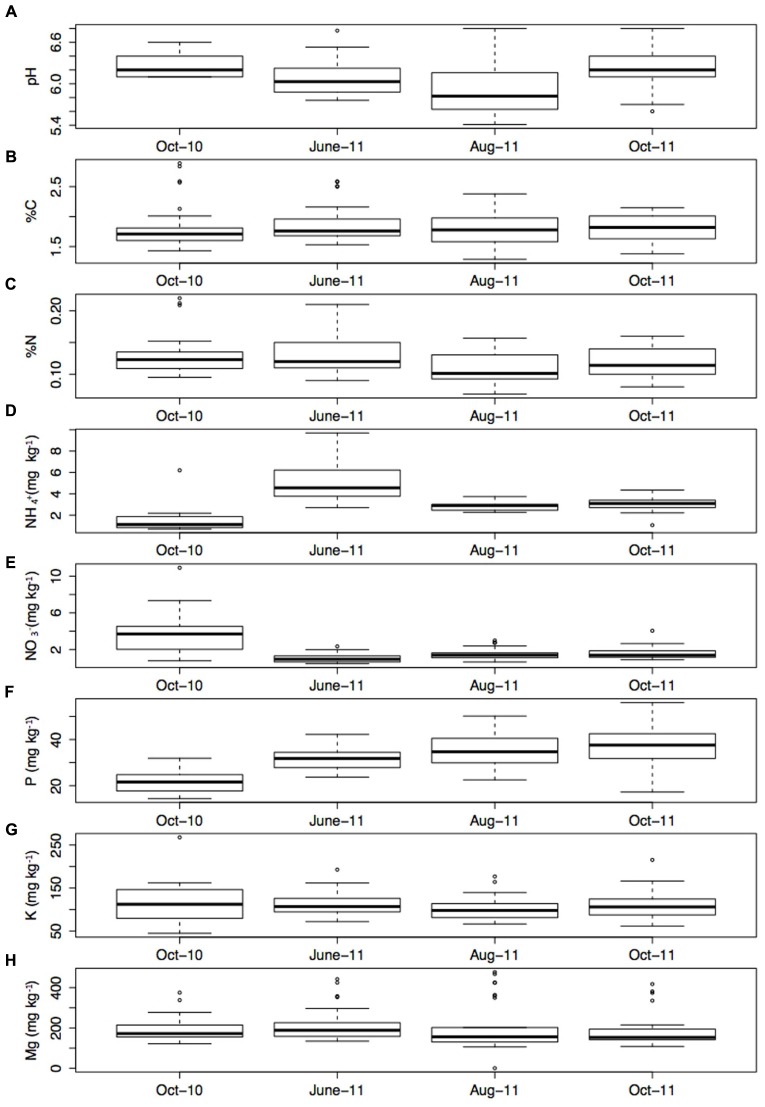
**(A)** pH, **(B)** % C, **(C)** % N, **(D)** NH_4_^+^ (mg kg^-1^), **(E)** NO_3_^-^ (mg kg^-1^), **(F)** P (mg kg^-1^), **(G)** K (mg kg^-1^), and **(H)** Mg (mg kg^-1^) at each sampling time point (the mean of four sub samples). Error bars represent 1 standard error of the mean.

Soil properties were stable over time (**Figure 3**), with the exception of NH_4_^+^ which was significantly higher in June-11 compared to the other sampling times (*P* < 0.001). All parameters showed variability within the transects. Soil pH varied between 5.41 and 6.80, and had a mean of 6.11. Meanwhile, K varied between 44.9 (mg/kg^-1^) and 267.7 (mg/kg^-1^) and had a mean of 199.7 (mg/kg^-1^). Soil C varied between 1.29 and 2.90% and had a mean of 1.83%, whilst %N ranged between 0.06 and 0.22% with a mean of 0.122%. Average Soil NH_4_^+^ content was 3.13 (mg/kg^-1^) and ranged between 0.71 (mg/kg^-1^) and 9.70 (mg/kg^-1^). Mg ranged between 0.9 and 476.3 (mg/kg^-1^), with an average of 198.8 (mg/kg^-1^), and finally P varied from 14.4 (mg/kg^-1^) to 56.0 (mg/kg^-1^) and had an average content of 31.5 (mg/kg^-1^).

### Root Length Colonization by Arbuscular Fungi

**FIGURE 4:**
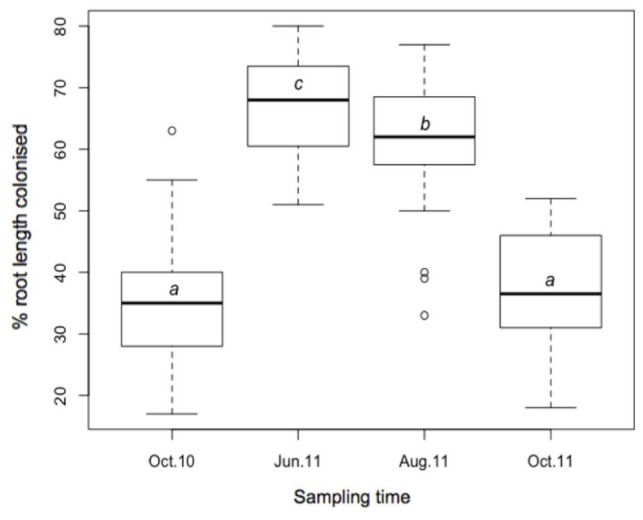
**Boxplot of AMF root length colonization; October-10, June-11, August-2011, and October-11, where *a, b*, and *c* are significantly different means (*t*-test)**.

Stained coils, arbuscules, hyphae, and spores were observed under a light microscope in all samples, confirming the presence of AMF in *M. giganteus* roots. RLC ranged from 17 to 80%, and varied across sampling times (**Figure 4**). Mean RLC was greatest in June-11 (68.1%) followed by August-11 (61.4%). Mean RLC of October 2010 and October 2011 was 34.9, and 37.4% respectively, which were both significantly lower than either of the summer time points (*P* < 0.001 for each comparison), but not significantly different to each other.

### Sequencing of the AMF Community

After hierarchical clustering, seven AMF OTUs were identified, with no non-AMF OTUs found (Supplementary Table S1). Six OTUs were present in each library. Sequences assigned to two *Glomus* OTUs were the most abundant, with 36.0 and 15.0% of sequences assigned to *Glomus* sp. *M09* in October-10 and June-11 respectively, whilst 27.9 and 36.3% of sequences were assigned to *Glomus* sp. *NBR8*. Further abundant sequences were related to *Paraglomus laccatum, Diversispora* sp. *EE1* and a *Glomeromycota* sp. *MIB8442* that were present in both sampling points, and accounted for an average of 23.3, 8.6, and 8.5% of total reads respectively over time. A further *Paraglomus laccatum* OTU was present in June-11, accounting for just 5% of reads, whilst an OTU related to *Glomeromycota* sp. *WR864* was found in October-10 only, and accounted for just 1.2% of reads.

### Core and Satellite Partitioning of AMF Communities

Twenty one TRFs were identified using TRFLP. Numbers of TRFs detected varied across time points, with averages of 11.0 in October-10 (ranging from 5 to 17 TRFs), 13.6 in June-11 (ranging from 8 to 18 TRFs), 13.5 in August-11 (ranging from 10 to 17 TRFs), and 13.7 in October-11 (ranging from 8 to 21 TRFs). October-10 had significantly lower TRF richness than June-11 (*P* < 0.001), August-11 (=*P* < 0.001), and October-11 (*P* < 0.001). There were no significant differences in TRF richness between the three sampling times in 2011. There was a positive log-normal relationship between persistence and abundance of TRFs at each sampling time (**Figure 2**). The number of core TRFs at each sampling time varied between 5 and 9, while there were between 8 and 16 satellite TRFs (Supplementary Figure S1). Five TRFs remained consistently at >75% persistence over time. A total of 15 of the 21 TRFs were considered persistent (core) in at least one time point. Across sampling times, the core AMF taxa represented 41.7% of TRF richness but 79.1% of TRF abundance.

### Spatio-Temporal Variation of AMF Communities

The 369 bp TRF was most abundant AMF within the community, with an average relative abundance of 16.3%, whilst other dominant core TRFs included 536 and 528 bp TRFs with average abundances of 12.3 and 10.8% respectively (**Table 1**). The relative abundance of many of TRFs was highly variable over time. For example the mean relative abundance of the 372 and 537 bp TRFs peaked in summer months, with the 372 bp TRF increasing from 0.4% in October-10, to 1.2% in June-11, 1.6% in August-11 before decreasing to 0.3% in October-11, whilst the 537 bp TRF rose from 2.8% in October-10, to 5.8% in June-11, 5.2% in August-11, before falling to 3.4% in October-11. Some TRFs remained relatively stable throughout the sampling period, such as the 369 bp TRF, which varied between 16.9 and 23.2%. There were six TRFs that were never core at any sampling time and also occurred in low abundance, ranging from a relative abundance of 0.1% to a maximum of 1.4%.

**Table 1:** Average relative abundance of each TRF at each sampling time.

	Mean relative abundance (%)			
TRF length (bp)	October-10	June-11	August-11	October-11
369	**18.9**	**16.9**	**22.4**	**23.2**
371	2.3	2	**3**	2.7
372	0.4	1.2	**1.6**	0.3
395	0.8	1.3	2.6	**11.4**
398	3.8	**5.5**	5.2	2.3
401	1.4	0.6	0.3	0.8
440	0.2	0	0.2	0.8
444	0.1	0.1	0.4	1.1
445	0.7	0.8	0.5	1.1
448	5.7	**6.5**	4.4	**7.6**
451	0.4	0.1	0.2	0.3
523	0.8	0.7	0.3	1
524	**7.4**	**5.6**	**6.5**	**6.8**
525	**8**	**5.1**	**5**	**5.9**
528	15.8	**9.7**	**10.9**	**6.9**
531	1.9	**7.3**	**3.8**	2.3
535	**4.4**	**7.2**	**7.4**	**4.8**
536	**12.3**	**13.6**	**12.3**	**9.7**
537	2.8	**5.8**	**5.2**	**3.4**
375	5.7	**4**	**2.9**	**1.8**
377	3.1	**2.9**	**1.8**	**2.6**

*Bold indicates TRF was part of the core community within the transect at the 75% threshold for persistence.*

**FIGURE 5:**
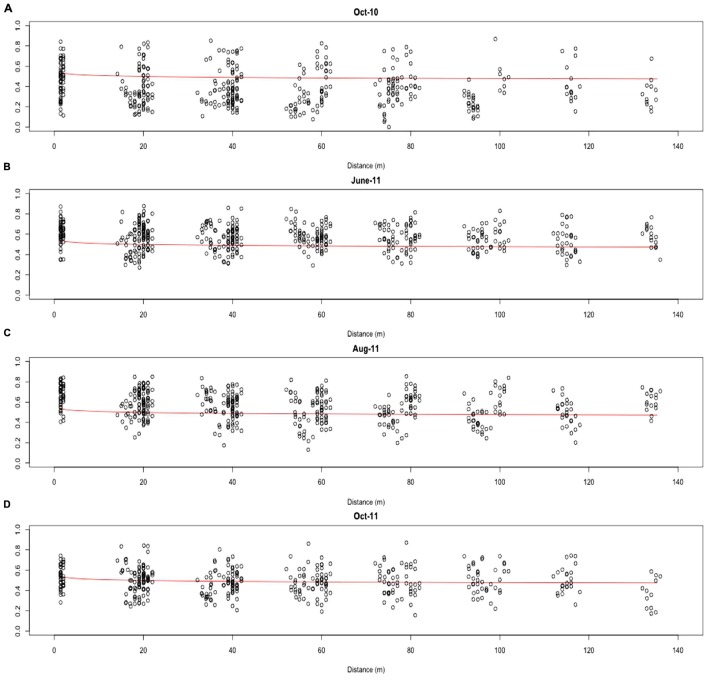
**Distance-decay of the combined AMF community similarity for (A) Oct-10, (B) June-11, (C) Aug-11 and (D) Oct-11.** Distance-decay values were calculated for the combined community using the formula *S* = *cD*^*ddr*^.

Distance-decay relationships were calculated to quantify β-diversity of the whole AMF community at each time point. Due to limited β-diversity, and particularly, low numbers of satellite community members, DDRs could not be determined for the core and satellite communities. The turnover of taxa was found to be consistently low across the whole 13 months sampling period, with decay values of just -0.061, -0.022, -0.058, and -0.027 for October-10, June-11, August-11, and October-11, respectively (Figure 5). Partial mantel statistics correlating community similarity to the distance matrix, whilst compensating for variation in soil nutrients, demonstrated significant independent geographical distance effects for both June-11 and October-11 (*R* = 0.195, *P* = 0.009 and *R* = 0.262, *P* = 0.002 respectively, i.e., communities closer together were more similar, even when differences in soil properties were taken into account), but not in October-10 and August-11 (*R* = 0.064, *P* = 0.231 and *R* = 0.003, *P* = 0.453 respectively).

Changes in the abundance and persistence of AMF TRFs were reflected in temporal shifts in whole, core and satellite community compositions (ANOSIM, *P* < 0.001 for each respectively). SIMPER analysis revealed that the whole community had an average similarity of 48.0% between time points, but varied between 40.9 and 56.4% (**Table 2**). The similarity between time points within the core community was considerably greater than either the whole or satellite communities, averaging 55.8% overall and ranging between 49.1 and 63.4%, whilst the satellite community had an average of just 28.6% community similarity between time points, ranging between 21.5 and 36.9%.

**Table 2:** Percentage similarity of whole, core and satellite AMF communities between sampling times.

	August-11	June-11	October-10	October-11
**Whole**				
August-11		56.4	45.2	50.7
June-11			45.4	49.6
October-10				40.9
**Core**				
August-11		63.4	51.7	59.7
June-11			51.7	59.2
October-10				49.1
**Satellite**				
August-11		36.9	27.2	29.6
June-11			28.8	27.9
October-10				21.5

### Regulation of Combined, Core, and Satellite Communities

The factors regulating the assembly of whole, core and satellite AMF communities at each time point were analyzed independently against soil properties (pH, C, N, NH_4_, NO_3_, P, K, Mg; **Table 3**). A sampling site effect (with each site consisting of the four subsamples) was also investigated using PERMANOVA. Due to strong autocorrelation, only cumulative variation was considered, as the community variation associated with individual soil properties could not be reliably disentangled. In October 2010, none of the variation in the whole and satellite communities could be explained, while 9.1% of variation in core communities could be attributed to soil properties. In contrast, in August-11, soil properties explained 54.5, 55.7, and 16.6% of variation in the whole, core and satellite communities respectively (**Table 3**). Meanwhile in June-11, soil properties explained 18.6, 20.7, and 9.9% of variation in the whole, core and satellite communities respectively. Similarly, variation in soil properties accounted for 12.1, 13.4, and 9.3% of variation in whole, core and satellite communities in October-11.

**Table 3:** Adonis analyses of the variance in of whole, core and satellite AMF communities explained by soil properties at each sampling time, and all times combined.

Community partitioning	October-10 (% variation explained)	June-11 (% variation explained)	August-11 (% variation explained)	October-11 (% variation explained)	All times (% variation explained)^∗^
Whole	0	18.67	54.53	12.11	25.63
Core	9.06	20.66	55.71	13.42	27.30
Satellite	0	9.86	16.64	9.30	21.81

*Due to spatial autocorrelation, values consist of the cumulative variation explained by soil parameters. ^∗^Indicates temporal variation was also included in the total percentage of variation.*

Data from across the four sampling times were combined, and cumulative variation attributed to time and soil properties was determined. The core community had a higher percentage of variation explained than either the combined or satellite communities (27.3, 25.6, and 21.8% respectively). The core component had the least percentage of variation explained by temporal variation at 3.9%, compared to 5.0 and 6.5% for the whole and satellite communities respectively.

## Discussion

In this work we show that within a monodominant *M. giganteus* plantation the AMF community was comprised of core and satellite taxa, the composition of which varied over time. However, composition of the core community showed less variation than the satellite community in both space and time over the 13 months sampling period. Furthermore a higher percentage of variation was explained by soil parameters at each time point for the core AMF community compared to that of either the satellite or whole communities. This greater spatio-temporal variation in the satellite community, coupled with less variation explained by measured parameters suggests a higher degree of stochasticity associated with satellite community assembly. However, it remains possible that the factors regulating the satellite community could reflect unmeasured parameters, such as climatic variables or interactions with other soil biota. In macro-organisms, Magurran and Henderson (2003) hypothesized that core species are well-adapted to their surroundings, whereas satellite species are under limitations of dispersal. We therefore suggest that the partitioning of AMF communities into core and satellite communities can be utilized to identify the community best suited to the local environment and which are also most likely to be regulated by it. Additionally, the ratio of relative abundance and richness of core and satellite community members may be a useful metric in describing community dynamics, providing insight into the degree of specialization and functional redundancy within microbial communities, yielding an approach that may ultimately improve the understanding of microbial community variation over space and time (van der Gast et al., 2011).

Whilst the core and satellite communities both showed differences in resilience to temporal variation, changes in the whole community were not purely driven by extinction and immigration of the satellite community, but largely matched dynamics of the core community. Similar results have been described before in the bacterioplankton within marine ecosystems (Lindh et al., 2016), in which only eight OTUs from a community of 1000s were persistent throughout an entire year sampling, with many OTUs core over only brief time periods. There was also considerable variation in the dynamics of individual taxa within the core and satellite communities over space and time. For example the core 372 bp TRF peaked in June-11 and August-11 whilst the core 401 bp TRF was lowest in the 2 October sampling points.

Declines in AMF RLC abundance matched the decline in TRF richness in the October-10 sampling points, but not in October-11, although it should, however, be noted that absent TRF are not necessarily extinct within the community but merely fall below the detection of the molecular analysis (Avis et al., 2006). Nonetheless There was a strong log-normal relationship between TRF persistence and abundance, as found in many population studies of macroorganisms and microorganisms (Hanski, 1982; Neubert et al., 2006; Dumbrell et al., 2011; Unterseher et al., 2011a,b). Partitioning of AMF communities into core and satellite communities provided similar persistence profiles to other studies of AMF fungi (Dumbrell et al., 2011). However, Unterseher et al. (2011a) found that AMF distribution did not fit a log-normal model, but did find that 40% of AMF taxa were found to be core, comprising of 95% of all OTUs. Although in our study AMF communities followed a log-normal core and satellite model, persistence profiles were comparable to Unterseher et al. (2011a), with 41.7% of taxa core, comprising 79.1% of AMF relative abundance.

Both soil properties and time played substantial roles in determining AMF community assembly. Variation in soil properties did have significant effects in regulating AMF communities, in agreement with an abundance of literature (Treseder et al., 2004; Cavagnaro et al., 2006; Gosling et al., 2013; Nouri et al., 2014). However, attempts to determine the edaphic properties regulating the AMF community were confounded by strong spatial autocorrelation between many of the properties. Whilst this is common in ecological studies, it is often not easily disentangled. Regardless, pH could be a significant factor in shaping the AMF community, having previously been linked to changes in AMF community composition of wild *Miscanthus sinensis* (An et al., 2008) and playing a near ubiquitous role in shaping microbial communities (Coughlan et al., 2000; Griffiths et al., 2011; Verbruggen et al., 2012). Geographical distance effects such as dispersal limitation have also been shown to influence fungal community assembly of other fungal groups at a local level (Green et al., 2004; Lilleskov et al., 2004; Peay et al., 2007; Barnes et al., 2016). Although, partial Mantel statistics suggest an independent distance effect within June-11 and October-11, β-diversity was exceptionally low across transects and studies of AMF biogeography at larger scales have found no evidence for geographical scaling (An et al., 2008; Hazard et al., 2013), suggesting very limited effects of dispersal limitation influencing the spatial scaling of AMF at the local level.

Both AMF community composition and the degree to which soil properties accounted for community variation were found to change substantially over the 13 months sampling period. RLC rates were highest in the summer months and lowest in the October sampling points, whilst October-10 also had significantly reduced TRF richness. Highest AMF colonization rates were found in the June-11 and August-11 samples, at peak growing times, before the onset of senescence for *Miscanthus* grown in temperate regions (Christian et al., 2008). This matches results from a number of previous studies in which seasonal variation in AMF communities have been suggested (Welsh et al., 2009; Tian et al., 2011; Dumbrell, 2013), although, further temporal replicates would be required to confirm this trend. The exact mechanism for temporal variation in AMF has yet to be fully determined. Importantly, soil properties remained relatively stable throughout the sampling period and could not explain temporal variation within the AMF community or changes in the influence of soil properties on AMF community composition between sampling times. Furthermore, the consistent sampling design of transects eliminated the possibility of geographical scaling effects driving the observed temporal variation within the AMF community. However, fluctuating carbon availability from host roots have been shown to influence the composition and abundance of root-associated microbes (de Neergaard and Gorissen, 2004; Jones et al., 2004), and can vary throughout the year (Saggar and Hedley, 2001) and with edaphic properties (Denef et al., 2009). Thus the observed spatial and temporal changes in AMF composition and RLC could reflect differences in exchange of resources between the symbionts.

## Conclusion

By partitioning communities into their core and satellite constituents we show that both the core and satellite communities vary spatially and temporally, and that local temporal variation within fungal communities is not only driven by variation within the infrequent, satellite community members. We also demonstrate that the removal of the more stochastic satellite community improved our ability to identify the factors regulating community assembly. Due to selective PCR biases, there is a continuing limitation of using data produced via metabarcoding techniques quantitatively (Pawluczyk et al., 2015; Piñol et al., 2015; Blanckenhorn et al., 2016), this approach can, however, be implemented on a presence/absence basis, as in this study, to further the understanding of factors determining microbial community regulation.

## Author Contributions

CJB analyzed data and wrote the manuscript. CAB participated in writing the manuscript and performed laboratory work. CG provided statistical and analytical support. NM contributed to the writing of the manuscript and provided key materials. GB is the senior author and designed experiments and provided funding. They also supervised research activities and participated in the writing of the manuscript.

## Conflict of Interest Statement

The authors declare that the research was conducted in the absence of any commercial or financial relationships that could be construed as a potential conflict of interest.
