# Supplementary material for: Spatio-Temporal Variation of Core and Satellite Arbuscular Mycorrhizal Fungus Communities in Miscanthus giganteus
Source: Front Microbiol. 2016 Aug 22;7:1278. doi: 10.3389/fmicb.2016.01278 (PMC4993019; doi:10.3389/fmicb.2016.01278)
Supplement: Supplementary file 1 [file Table_1.DOCX]

**Supplementary Tables**

**Supplementary Table S1** Clone library sequences isolated from the Oct-10 and Jun-11 sampling points were there highest taxonomic assignments.

| **Accession numbers** | **Assigned Taxonomy** | **Query Coverage** | **Identity** | **Oct-10 (% of clones)** | **Jun-11 (% of clones)** |
| --- | --- | --- | --- | --- | --- |
| KU937164 | *Glomus sp. M09* | 99% | 96% | 36.0 | 15.0 |
| KU937170 | *Glomus sp. NBR8.7* | 99% | 97% | 27.9 | 36.3 |
| KU937161 | *Paraglomus laccatum* | 100% | 99% | 22.1 | 22.5 |
| KU937154 | *Diversispora sp. EE1* | 100% | 99% | 5.8 | 11.3 |
| KU937168 | *Glomeromycota sp. MIB 8442* | 100% | 97% | 7.0 | 10.0 |
| KU937155 | *Paraglomus laccatum* | 100% | 99% | 0.0 | 5.0 |
| KU937150 | *Glomeromycota sp. WR864-A* | 100% | 96% | 1.2 | 0.0 |

**Supplementary Figures**

**Supplementary Figure 1** Line graph of TRF persistence (%) over time, with shape representing core (core in all time points), intermittent (core in some time points) and satellite (satellite in all time points) TRFs.
